# Supplementary material for: Physiology of PNS axons relies on glycolytic metabolism in myelinating Schwann cells
Source: PLoS One. 2022 Oct 4;17(10):e0272097. doi: 10.1371/journal.pone.0272097 (PMC9531822; doi:10.1371/journal.pone.0272097)
Supplement: S1 File — (DOCX) [file pone.0272097.s001.docx]

**SUPPLEMENTARY MATERIALS:**


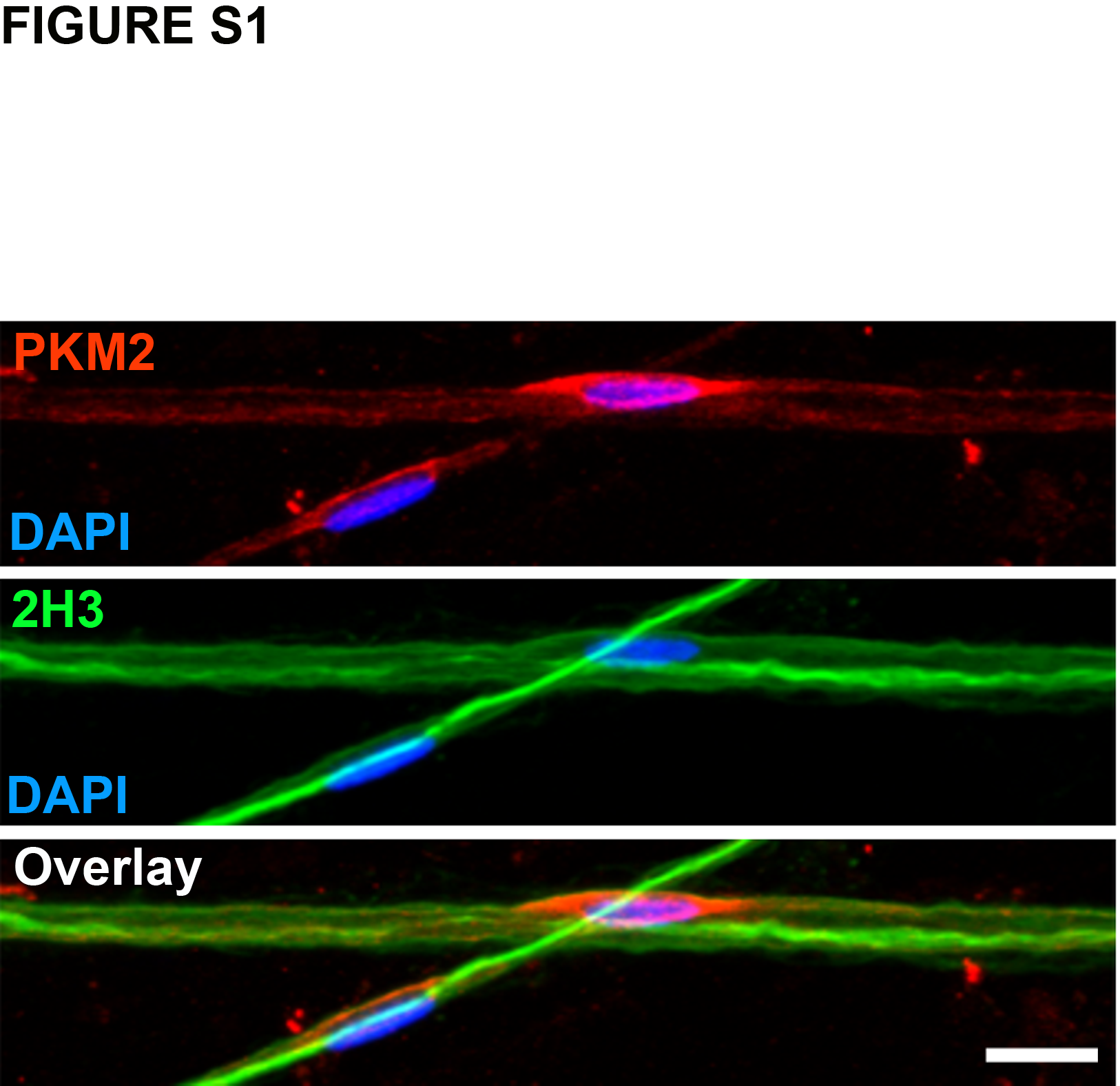


**FIGURE S1 PKM2 is enriched in the perinuclear cytoplasm of mSC.**

Immunostaining of mature mSC in teased mouse sciatic nerve (1 month old) fibres for PKM2, axonal 2H3 and with nuclear DAPI show the enrichment of PKM2 in the perinuclear cytoplasm of mSC.

Scale bar= 10μm.

**
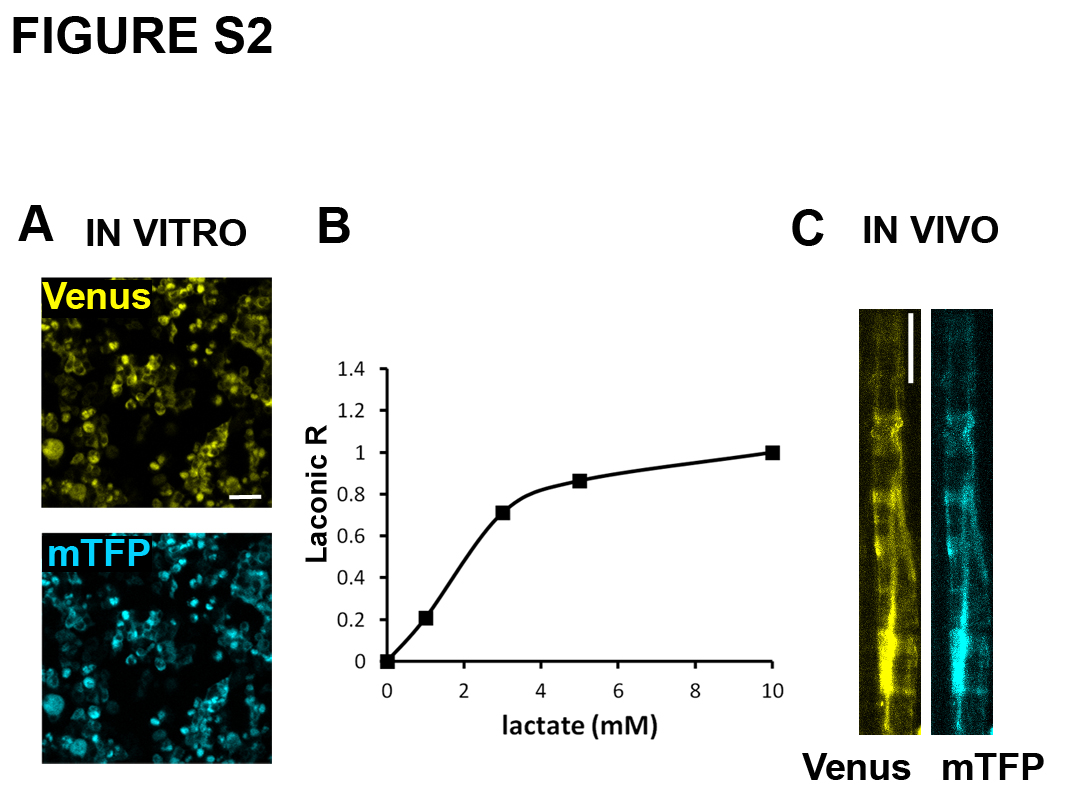
**

**FIGURE S2 PKM2 is enriched in the perinuclear cytoplasm of mSC.**

Immunostaining of mature mSC in teased mouse sciatic nerve (1 month old) fibres for PKM2, axonal 2H3 and with nuclear DAPI show the enrichment of PKM2 in the perinuclear cytoplasm of mSC.

Scale bar= 10μm.

**FIGURE S3 Biochemical measure of lactate in frontal part of mouse brains.**

N= 5 animals per condition. Two-tailed Student t-test Pvalue= 0.76. AU: arbitrary unit. Error bars represent SEM.

**
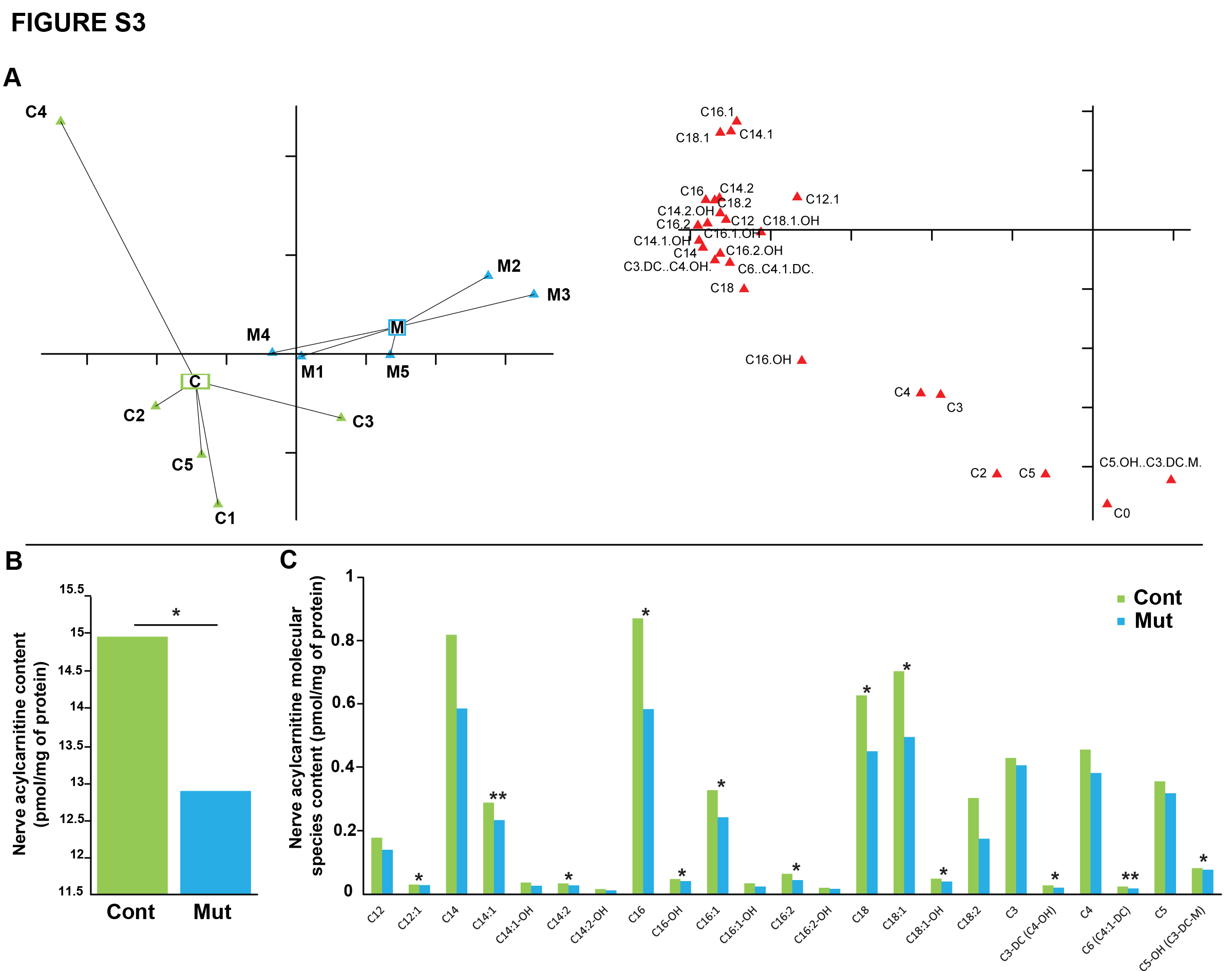
**

**FIGURE S4 Metabolomic analysis of Control and Mutant sciatic nerves. A**- A PCA performed on acylcarnithines separates Mutant (M) from Control (C) mouse nerves with strong eigenvalues (horizontal axis 64% and vertical axis 17%). Mutant and Control are mainly separated along the axis-1 showing that Control are associated with stronger occurrences of long chain acylcarnithines. n=5 male mice per genotype. **B**- Nerves of Mutant mice contain significantly less acylcarnitines than Controls. **C-** The systematic analysis of acylcarnitines shows a significant decrease of long-chain molecular species in Mutant vs Control nerves.

n=5 male mice per genotype. Female metabolism was noisier due to hormonal variability, so we excluded female mice from the analysis. Statistical test show paired two-tailed Student T-test. * = P<0.05.


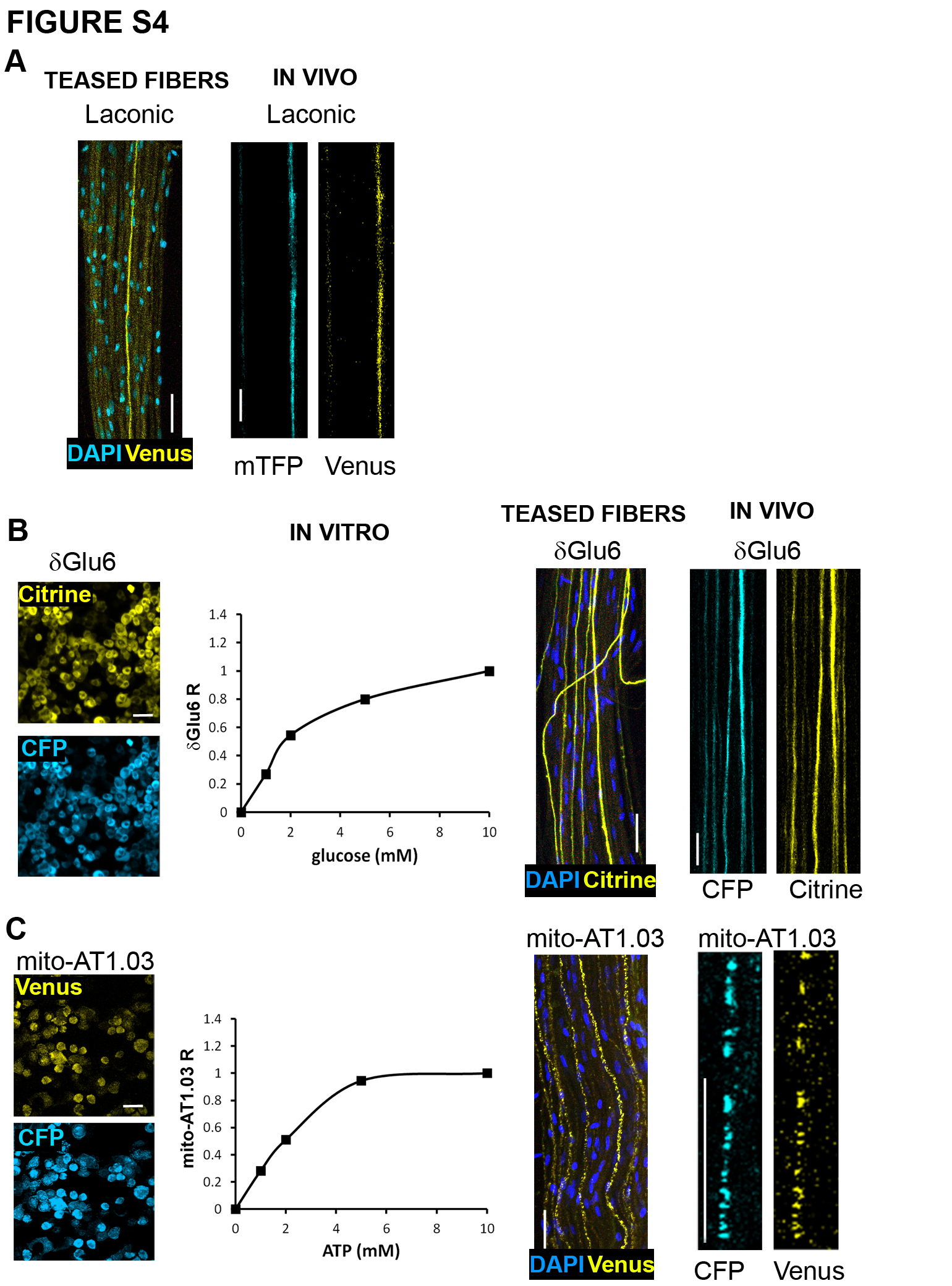


**FIGURE S5 Functional validation of the fluorescent probes. A- left:** HEK293 cells were transfected with pAAV-Laconic and imaged for Venus (yellow) and mTFP (blue)(scale bar= 50 μm). Then Venus/ mTFP fluorescence ratio (Laconic R) was measured at different concentration of lactate (0, 1, 3, 5, 10 mM) showing a correlation between increasing amount of lactate and Laconic fluorescence ratio (Graph). **Right:** AAV9 expressing Laconic under a CAG promoter was injected in the spinal cord of newborn mice and one month later Mutant and control animals were treated with Tamoxifen. Three to five weeks later, sciatic nerve fibers were teased on a glass slide. Laconic (Venus, yellow) is expressed in long never-ending axons (scale bar= 100μm). DAPI (blue) shows nuclei of the surrounding cells. In addition, the saphenous nerve of anesthetized mice expressing Laconic was exposed under the lens of a multi-photon microscope and both Venus (yellow) and mTFP (blue) fluorescence were recorded in order to measure Laconic fluorescence ratio *in vivo* (scale bar= 10μm). **B**- Similar experiments were done as in **A** for δGlu6 fluorescent probe. δGlu6 fluorescence ratio (Citrine/CFP) is proportional to glucose concentration (0, 1, 2, 5, 10mM). The probe is expressed in axons crossing the sciatic nerve and the exposed saphenous nerve allows to record this ratio in axons of living anesthetized mice. **C**- Similar experiments were done as in **A** for mito-Ateam (mito-AT1.03). Mito-Ateam fluorescence ratio (Venus/CFP) is proportional to ATP concentration (0, 1, 2, 5, 10mM). The probe is expressed in mitochondria (dotted pattern) of axons crossing the sciatic nerve and the exposed saphenous nerve allows to record this ratio in axonal mitochondria of living anesthetized mice.


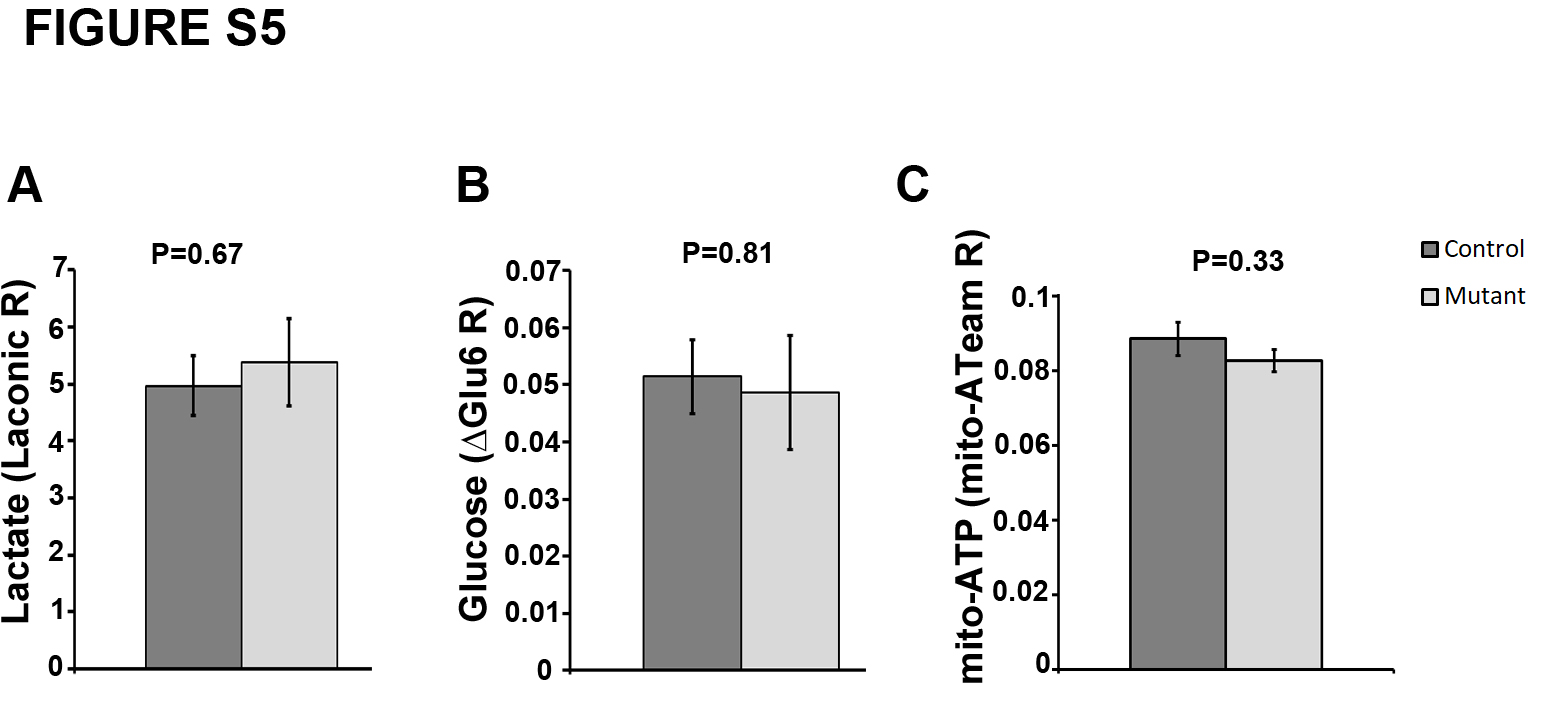


**FIGURE S6 Lactate, glucose and mitochondrial ATP levels in resting axons of Mutant and Control saphenous nerves are not different.** As described in Figure S3, Laconic (**A**), δGlu6 (**B**) and mito-Ateam (**C**) fluorescence ratios were measured in resting axons of the saphenous nerves of Mutant and Control mice. No significant differences could be detected in these conditions. P-values show two-tailed unpaired Student T-test. A: n=15 axons in 7 Control mice, 7 axons in 4 Mutant mice. B: n=9 axons in 3 Control mice, 6 axons in 3 Mutant mice. C: n=14 axons in 5 Mutant mice, 16 axons in 5 Control mice. Error bars represent SEM.

**FIGURE S7 Open Field (OPF) test shows no difference between Mutant and Control mice.**

Mutant and Control mice were tested for their CNS functions using Open Field test. The covered distance (OPF distance), the mean velocity to cover this distance (OPF mean velocity) and the resting time (OPF resting time) were measured. N=10 (Control) and 8 (Mutant). Error bars show SEM. P-values show two-tailed unpaired Student T-test.


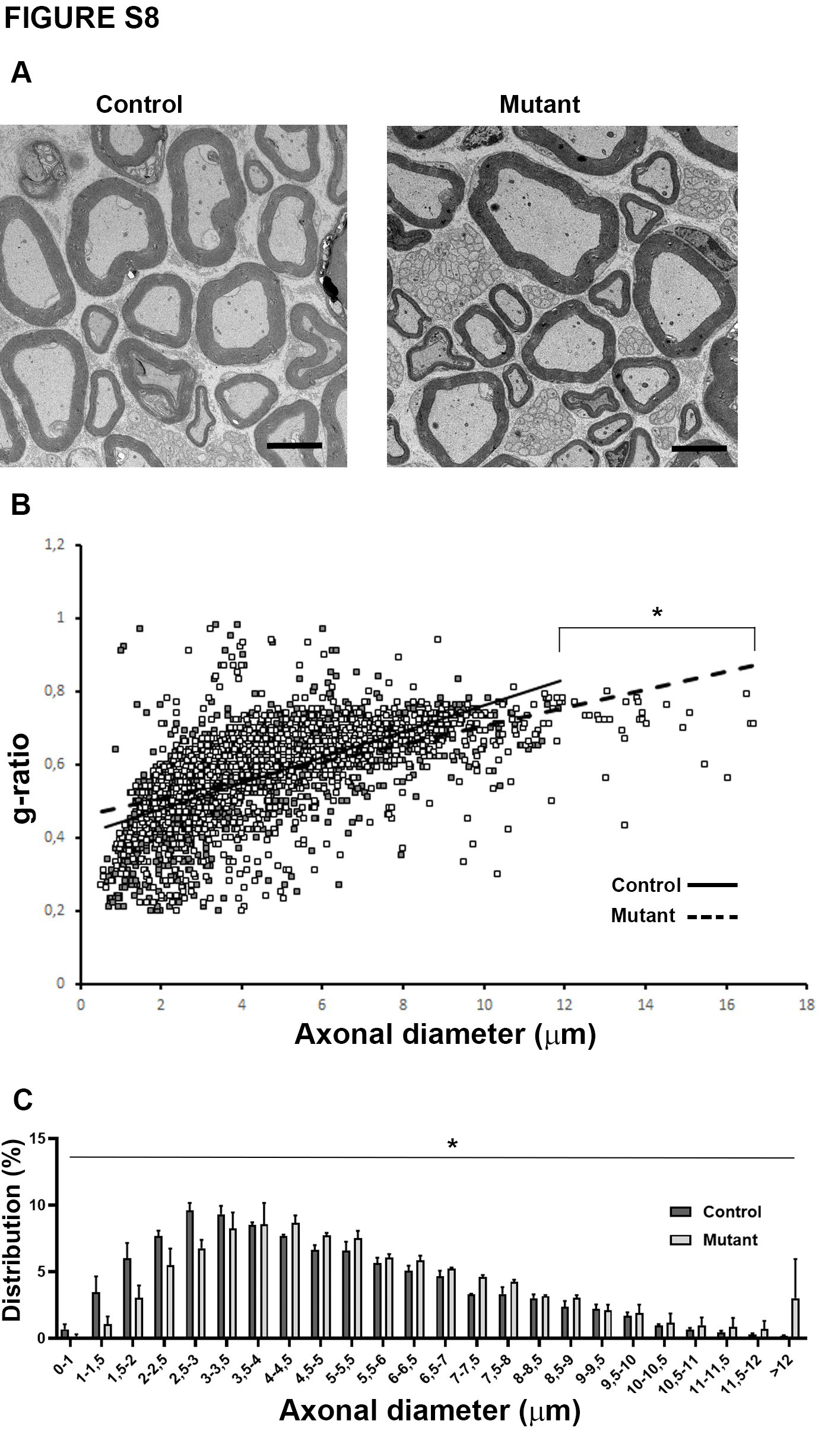


**FIGURE S8 Electron microscopy analysis of Mutant and Control mouse sciatic nerves.** **A**- Ultrathin sections of Control and Mutant mouse sciatic nerves (12 months old) did not reveal any structural difference between genotypes. Scale bars= 4μm. **B**- Using semi-thin section, the ratio axon diameter on full fiber diameter (g-ratio) was measured and plotted relative to the axon diameter. Statistical unpaired two-tailed Student T-test showed a slight significant increase in mutant g-ratio (0.56 vs 0.59, P-value <0.001). **C**- Axonal diameter distribution was plotted over axonal diameter category. Statistical two-way ANOVA analysis followed by a Sidak multiple comparison test comparing mean value for each axonal diameter category showed no significant difference between Control and Mutant mice (genotype P-value = 0.95; see raw data file). However, an overall significant interaction was found between genotype and axonal diameter category (interaction P-value = 0.017; see raw data file). N= 3 animals for each genotype and a total of 1593 and 1256 fibers in Control and Mutant genotype respectively.


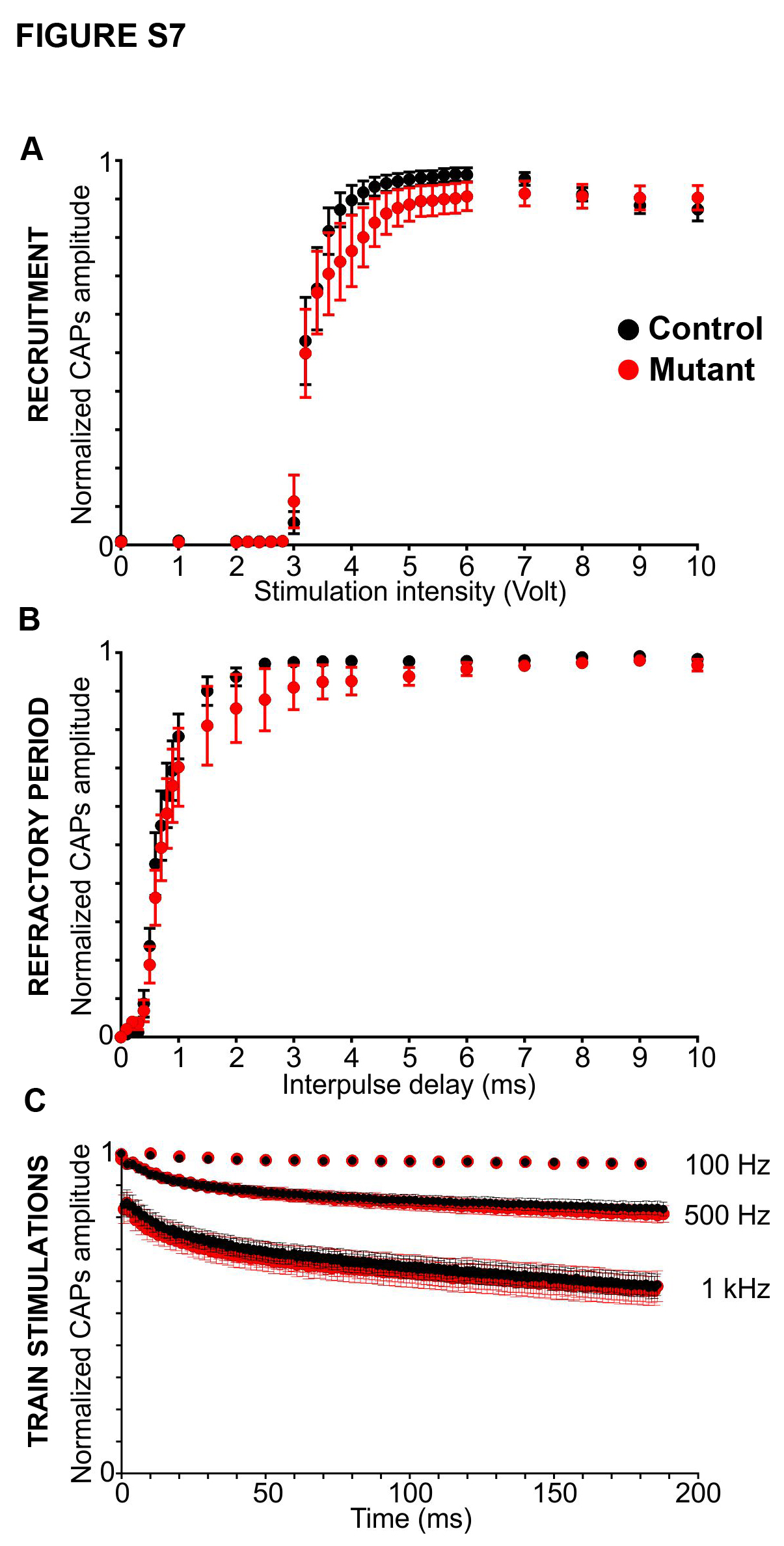


**FIGURE S9 Electrophysiological analysis of Control and Mutant sciatic nerves show no significant alteration of the action potential propagation along axons. A, B**- The recruitment (**A**) and refractory period (**B**) of sciatic nerves from Mutant mice (n = 10 nerves from 5 mice) is not significantly different from those of Control mice (n = 10 nerves from 5 mice) (P>0.05 by two-tailed t-tests for two samples of equal variance). **C**- Nerves were stimulated with train of stimuli ranging from 100 to 1 kHz in order to monitor the sustainability of the response. No difference was observed between Control and Mutant mice. Error bars represent SEM.


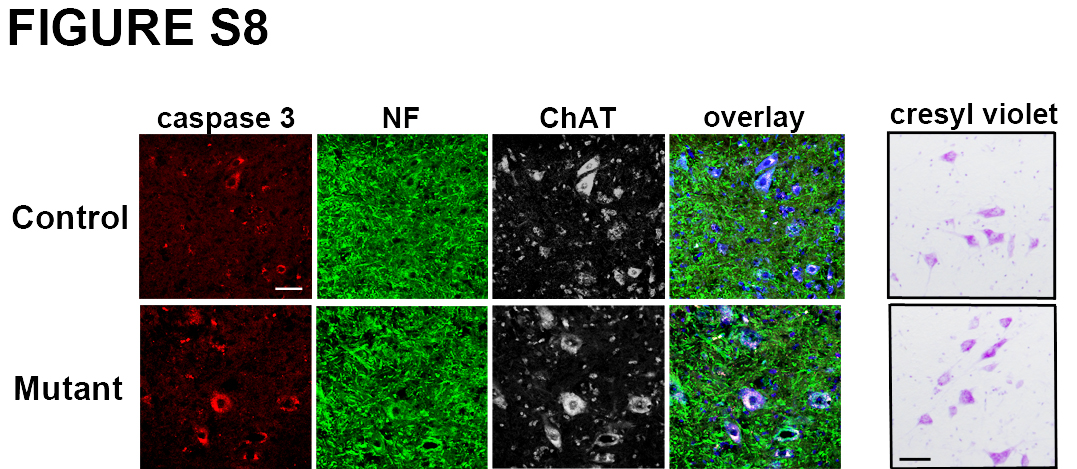


**FIGURE S10 Mutant mouse motor neurons display neuronal stress marker Caspase 3.** Spinal cord cryosections of Mutant and Control mice were stained with cresyl violet or immunostained for neuronal stress marker Caspase 3 (red), neuronal marker Neurofilament (NF, green) and motor neurons marker ChAT (blue). While Neurofilament staining is not different, more motor neurons express Caspase 3 in Mutant mice than in Control mice. Scale bars= 50μm.
