# Supplementary material for: Physiology of PNS axons relies on glycolytic metabolism in myelinating Schwann cells
Source: PLoS One. 2022 Oct 4;17(10):e0272097. doi: 10.1371/journal.pone.0272097 (PMC9531822; doi:10.1371/journal.pone.0272097)
Supplement: S1 Table — (DOCX) [file pone.0272097.s002.docx]

**TABLE S Electrophysiological characteristics of Control and Mutant mice.**

|  | Control | Mutant |
| --- | --- | --- |
| Amplitude (mV) | 2.5 ± 1.8 | 3.2 ± 2.5 |
| Duration (ms) | 0.34 ± 0.06 | 0.37 ± 0.09 |
| CV_V½_ (m.s^-1^) | 52.1 ± 15.8 | 45.2 ± 16.1 |
| CV_Vmax_ (m.s^-1^) | 33.8 ± 8.5 | 30.6 ± 9.4 |
| n | 10 (5 animals) | 10 (5 animals) |
