# Supplementary figures and images for: Physiology of PNS axons relies on glycolytic metabolism in myelinating Schwann cells
Source: PLoS One. 2022 Oct 4;17(10):e0272097. doi: 10.1371/journal.pone.0272097 (PMC9531822; doi:10.1371/journal.pone.0272097)

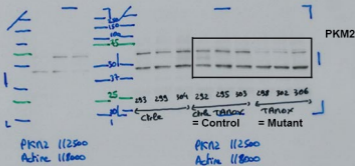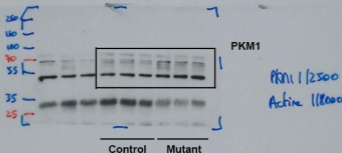

105 24/08/17.

Supplement: S1 Raw images — (PDF) [file pone.0272097.s004.pdf]
