# Supplementary material for: Physiology of PNS axons relies on glycolytic metabolism in myelinating Schwann cells
Source: PLoS One. 2022 Oct 4;17(10):e0272097. doi: 10.1371/journal.pone.0272097 (PMC9531822; doi:10.1371/journal.pone.0272097)
Supplement: Attachment — Submitted filename: Response to reviewer comments3.docx [file pone.0272097.s006.docx]

Responses to the reviewer comments

Reviewer #1: The authors have addressed all my concerns. I understand the difficulty of performing the lactate rescue experiments and including Mpz-cre.

Reviewer #2: The authors have made a good effort to address the concerns of the reviewers by amending text and adding new data and the manuscript is much improved. I have a couple of points that should be addressed.
1. In response to a request for comment on prior reports of MNCV slowing in DCA-exposed rodents the authors write “Over the seven weeks of treatment, the chosen dose (500mg/kg daily) was not sufficient to induce the significant reduction of nerve conduction velocity (Fig. 4C) reported previously (36,37), which suggests that nerve conduction is less dependent on mSC lactate in adult rats than in juvenile rats”. This interpretation is not correct as a) reference #37 reports MNCV slowing in adult DCA-exposed mice, not rats and b) reference #36 shows MNCV slowing in adult rats but not juvenile rats after exposure to 500mg/kg. DCA. The authors could simply note that their present MNCV data in adult mice exposed to 500mg/kg DCA does not replicate the prior report (ref #37) that used 1g/kg of DCA in adult mice – perhaps simply due to the lower dose used (note that 500mg/kg in rats as per reference #36 ≠ 500mg/kg in mice due absence of allometric scaling, whereas 500mg/kg in rats = 1g/kg in mice after allometric scaling).

*We agree with the reviewer and we modified the results section page 20 line 430 as following: “Over the seven weeks of treatment, the chosen dose (500mg/kg daily) was not sufficient to induce the significant reduction of nerve conduction velocity (****Fig. 4C****) reported previously at 1g/kg (36). Nevertheless, Rotarod performance decreased in treated mice independently of the genotype (****Fig. 4B****), suggesting an axonal defect.”*

2. The authors provide an interesting axonal size:frequency histogram (Fig S8C) and indicate that there was no difference between control and DCA exposed rats using an unpaired t test. I do not understand this analysis – what data were compared? The paper cited in the response to review used unpaired t test to compare mean axonal diameter, not the size:frequency histogram. Please report values (mean±SD) for MAD in the 2 groups to support any statistical analysis. It is also necessary to report fibers/nerve in order to fully interpret MAD or fiber size:frequency distributions, not just total number of fibers counted.

*Raw data including data relative to results presented in Figure S8C are shown in the “raw data” file. We agree with the reviewer that the Student T-test comparing the overall mean of axonal diameter is not accurate. We propose to use a two-way ANOVA test comparing genotypes in all axonal diameter category with a multiple analysis in each category. This analysis was done with Graphpad Prism and is presented in detail in the raw data file for Figure S8C.*

*We found that, while no significant difference can be found between genotypes regarding each axonal diameter category, there is an overall significant interaction between axonal diameter and genotype. This was noted in the Figure S8C legend : “ Statistical two-way ANOVA analysis followed by a Sidak multiple comparison test comparing mean value for each axonal diameter category showed no significant difference between Control and Mutant mice (genotype P-value = 0.95; see raw data file). However, an overall significant interaction was found between genotype and axonal diameter category (interaction P-value = 0.017; see raw data file).” In addition, we noted this in the results section of the manuscript page 19 line 410: “Axonal diameter distribution was also slightly shifted toward larger axons in Mutant vs Control animals (****Fig. S8C****). As larger axons display a higher g-ration (Fig. S8A), this change may explain the g-ratio increase. However, the conduction of action potentials along myelinated axons was not altered as the electrophysiological properties of sciatic nerve axons ex vivo were maintained even at very high firing frequencies (****Fig. S9, Table S****).”*

*A comment about the statistical analysis was added to the Material and Methods section page 15 line 325: “****Statistical analysis*** *Except indicated otherwise, statistical analysis were done using Graphpad Prism (GraphPad Software, San Diego, USA). Statistical significance: *= P value<0.05; **= P value<0.01; ***= P value<0.001.”*

Given that the size:frequency histogram (Figure S8C) suggests that DCA-exposed nerve may have a shift towards increased frequency of larger axons and reduced frequency of smaller axons, to what extent does increased axonal MAD contribute to the reported increase in g-ratio rather than the myelin thinning that the authors infer?

*We indeed suggested that the decrease in the g-ratio may result from the shift of axon diameter distribution toward larger axons and therefore higher g-ratio.*

Reviewer #3: The authors have addressed my concerns. I have no additional concerns about the research ethics or publication ethics. This is an interesting study which adds knowledge to the field
